# Artificial Intelligence Methods Applied to Electronic Health Record Data for Health Equity in Clinical Trials

**DOI:** 10.1016/j.jacadv.2025.102201

**Published:** 2025-10-08

**Authors:** Jonathan Breeze, Jack Wu, Josie Carmichael, Nilesh Pareek, Richard JB. Dobson, Ajay M. Shah, Kevin O'Gallagher

**Affiliations:** a King's College Hospital NHS Foundation Trust, London, United Kingdom; b School of Cardiovascular and Metabolic Medicine & Sciences, British Heart Foundation Centre of Research Excellence, King's College London, London, United Kingdom; c Moorfields Eye Hospital NHS Trust, London, United Kingdom; d Department of Biostatistics and Health Informatics, Institute of Psychiatry, Psychology and Neuroscience, King's College London, London, United Kingdom

**Keywords:** clinical trial representation, coronary artery disease, natural language processing, patient diversity, socioeconomic factors

For randomized controlled trials (RCTs) to provide relevant information to guide clinical practice, it is vital that the characteristics of the recruited trial cohort reflect those of the underlying disease population. If the recruited cohort is not representative of the underlying population, selection bias may limit the real-world effectiveness of the intervention.^1^, ^2^, ^3^ In particular, if RCT populations are skewed in terms of race, ethnicity, and social deprivation, there is potential to exacerbate existing health disparities.

**What is the clinical question being addressed?**

Are patients recruited into CAD RCTs representative of the real-world population?

**What is the main finding?**

RCT participants were less comorbid and included fewer women, minorities, and socioeconomically disadvantaged patients.

Natural language processing (NLP) has emerged as a powerful artificial intelligence–based method in the field of health care, offering the ability to efficiently analyze vast amounts of both structured and unstructured clinical data.

In this proof-of-concept study, we leveraged the capabilities of NLP to evaluate the representativeness of patients recruited to coronary artery disease (CAD) RCTs at our center by comparing them to the real-world CAD population from our hospital's electronic health record (EHR). We sought to uncover disparities and ensure that RCTs are as inclusive and representative as possible.

## Methods

This retrospective, observational study operated under London South-East Research Ethics Committee approval (18/LO/2048) granted to the King's Electronic Records Research Interface. The study complies with the Declaration of Helsinki. We identified patients who had been enrolled in 17 CAD RCTs at King's College London NHS Foundation Trust, UK—a large specialist cardiac center and teaching hospital serving a diverse urban patient population—between 2012 and 2023 by searching for the SNOMED code: 53741008. The overall CAD population under care (“real-world population”) was identified using our center's well-validated EHR NLP pipeline based on CogStack^4^ (for information retrieval) and MedCAT^5^ (for concept annotation), enabling the integration and analysis of structured and unstructured data, allowing comprehensive cohort characterization. For statistical comparisons, we used *t*-tests, Mann-Whitney *U* tests, and chi-square tests for continuous (normal and non-normal) and categorical variables, respectively.

## Results

We identified 20,066 patients with a diagnosis of CAD within the “real-world population.” Of these, 722 were recruited into CAD RCTs (“RCT cohort”). Both groups had a mean age of 65 years old; however, compared to the real-world population, the RCT cohort had lower proportions of females (20.9% vs 36.1%; *P* < 0.001); patients of Black or Asian ethnicity (11.8% vs 20.4%; *P* < 0.001); and patients in the lowest tertile of socioeconomic deprivation as defined by the index of multiple deprivations (14.0% vs 24.7%; *P* < 0.001), see Table 1. Patients in the RCT cohort had fewer comorbidities than the real-world population (median: 0.0 [IQR: 0.0-2.0] vs 1.0 [IQR: 1.0-3.0]; *P* < 0.001) with lower incidence of cardiovascular-related comorbidities such as diabetes (14.3% vs 22.2%; *P* < 0.001), hypertension (34.3% vs 45.8%; *P* < 0.001), atrial fibrillation (6.2% vs 11.2%; *P* < 0.001), and stroke/transient ischemic attack (8.2% vs 15.1%; *P* < 0.001). Smoking history was similar between groups (71.5% vs 71.0%; *P* = 0.8). Patients in the RCT cohort had fewer hospitalizations than the real-world cohort (2.4 ± 2.7 vs 3.6 ± 5.0; *P* = 0.001).

**Table 1:** Demographic and Comorbidity Data of RCT Cohort and Real-World Population

	RCT Cohort (n = 722)	Real-World Population (n = 20,066)	*P* Value
Demographics			
Age, y, mean (SD)	65.2 (11.1)	65.4 (13.6)	0.658
Sex, n (%)			
Female	151 (20.9)	7,244 (36.1)	<0.001
Male	571 (79.1)	12,822 (63.9)	
Ethnicity, n (%)			
White	425 (58.9)	12,563 (62.6)	<0.001
Black	44 (6.1)	2,544 (12.7)	
Asian	41 (5.7)	1,539 (7.7)	
Most socioeconomically deprived (lowest IMD tertile), n (%)	101 (14.0)	4,963 (24.7)	<0.001
Least socioeconomically deprived (highest IMD tertile), n (%)	116 (16.1)	3,907 (19.5)	0.026
IMD, mean (SD)	5.7 (2.7)	5.4 (2.6)	0.016
Comorbidities			
Type 1 diabetes, n (%)	18 (2.5)	898 (4.5)	0.014
Type 2 diabetes, n (%)	86 (11.9)	3,550 (17.7)	<0.001
Hypertensive disorder, n (%)	248 (34.3)	9,186 (45.8)	<0.001
Stroke, n (%)	39 (5.4)	2,051 (10.2)	<0.001
Transient ischemic attack, n (%)	20 (2.8)	983 (4.9)	0.011
Atrial fibrillation, n (%)	45 (6.2)	2,255 (11.2)	<0.001
Pulmonary hypertension, n (%)	20 (2.8)	491 (2.4)	0.668
Kidney disease, n (%)	51 (7.1)	2,889 (14.4)	<0.001
Liver disease, n (%)	21 (2.9)	1,506 (7.5)	<0.001
Never smoked, n (%)	206 (28.5)	5,827 (29.0)	0.800
Number of comorbidities, median [IQR]	0 [0-2]	1 [1-3]	<0.001

IMD = Index of Multiple Deprivations; RCT = randomized controlled trial.

## Discussion

We used NLP to identify our center's population of CAD patients to allow comparison with those patients recruited into CAD clinical trials. We find that there are significant differences in the demographics and comorbidity profile of patients recruited to RCTs compared to the underlying real-world CAD population. Females, patients from ethnic minorities, and those from areas of high socioeconomic deprivation were less likely to be recruited into RCTs. The RCT cohort was less comorbid and experienced fewer hospitalizations than the real-world population, suggesting in broad terms that the RCT cohort is healthier and less complex than the real-world population. While RCTs may purposefully include selected populations for scientific purposes, it is more likely that our results reflect biases in participant recruitment, which has an impact on generalization of RCT findings in clinical practice.

We anticipate that, with validation, this study's NLP-based methods could be used to develop EHR-based tools to assist in the reporting of the representativeness of a clinical trial's population compared to the underlying real-world cohort, both at an individual center- and trial-wide level. Moreover, such methods could be used in a prospective manner, for example, integrated EHR-based apps to highlight patients suitable for clinical trials and to alert recruiting clinicians if the RCT cohort deviates in characteristics from the real-world population, however, we appreciate there would be issues with prospective consent that would need to be overcome in this strategy.

The use of NLP is a strength of this study, allowing automated identification of large patient cohorts. A further strength is the ability to adopt this approach to any medical disease, that is, this is not a cardiology-specific approach. This study has several limitations. The first limitation is its single-center nature which is compared to the multicenter nature of most large RCTs. Second, due to its retrospective nature, the findings are only hypothesis-generating. Third, we did not include individual RCT details which may limit interpretability. Finally, the real-world population may include less rigorously confirmed diagnoses than the RCT cohort. Further work will be performed in other cardiology centers to test whether our findings are consistent across settings and explain the underrepresentation of particular demographic groups in recent large RCTs.

## Conclusions

In summary, we find that RCT cohorts are not representative of the real-world CAD population which potentially introduces bias into the trial methodology. Our methods can be used to develop tools to address this important health equity issue.

## Funding support and author disclosures

Dr O'Gallagher is supported by a Medical Research Council Clinician Scientist Fellowship (MR/Y001311/1). Dr Dobson is the co-founder of CogStack. Dr Shah has served as an advisor to Forcefield Therapeutics and CYTE-Global Network for Clinical Research. All other authors have reported that they have no relationships relevant to the contents of this paper to disclose.
